# Comprehensive coverage of human last meal components revealed by a forensic DNA metabarcoding approach

**DOI:** 10.1038/s41598-021-88418-x

**Published:** 2021-04-23

**Authors:** Judith Schneider, Eduard Mas-Carrió, Catherine Jan, Christian Miquel, Pierre Taberlet, Katarzyna Michaud, Luca Fumagalli

**Affiliations:** 1 Laboratory for Conservation Biology, Department of Ecology and Evolution, Biophore, University of Lausanne, 1015 Lausanne, Switzerland; 2 University Center of Legal Medicine Lausanne and Geneva, Lausanne University Hospital (CHUV) and University of Lausanne, Ch. de la Vulliette 4, 1000 Lausanne 25, Switzerland; 3 Laboratoire d’Ecologie Alpine, CNRS, Université Grenoble Alpes, 38000 Grenoble, France; 4 UiT, The Arctic University of Norway, Tromsø Museum, Tromsø, Norway

**Keywords:** Ecology, Biodiversity, Molecular ecology, Next-generation sequencing, Public health

## Abstract

Stomach content analyses are a valuable tool in human forensic science to interpret perimortem events. While the identification of food components of plant and animal origin has traditionally been conducted by macro- and microscopical approaches in case of incomplete digestion, molecular methods provide the potential to increase sensitivity and taxonomic resolution. In particular, DNA metabarcoding (PCR-amplification and next generation sequencing of complex DNA mixtures) has seen a rapid growth in the field of wildlife ecology to assess species’ diets from faecal and gastric samples. Despite clear advantages, molecular approaches have not yet been established in routine human forensics to investigate the last meal components of deceased persons. In this pilot study we applied for the first time a DNA metabarcoding approach to assess both plant and vertebrate components of 48 human stomach content samples taken during medicolegal autopsies. We obtained a final dataset with 34 vertebrate and 124 vegetal unique sequences, that were clustered to 9 and 33 operational taxonomic units (OTUs), respectively. Our results suggest that this approach can provide crucial information about circumstances preceding death, and open promising perspectives for biomedical dietary surveys based on digested food items found in the gastrointestinal tract.

## Introduction

Postmortem stomach content analyses are an essential tool in forensic science. In addition to e.g. pathological or toxicological investigations, the identification of organic material of plant and animal origin may give valuable information not only about the last meal components but also the last hours surrounding death and its time-frame, as well as establishing a link between a victim and a suspect or a location^1–3^. Macroscopic and microscopic inspection is the standard method to morphologically identify food items found in the stomach of deceased persons when autopsies are performed. However, this approach is of limited efficiency especially if food components have been rendered non-identifiable due to chewing and the digestive processes occurring in the highly acidic environment of the stomach. In addition, the structure of food items can be too similar between different taxa to allow unambiguous taxonomic identification.

Over the last decade, molecular methods have increasingly been employed to study the diet components of several non-human organisms, due to the advances in DNA amplification and sequencing technologies. In particular, DNA metabarcoding (i.e. the simultaneous PCR-amplification with universal primers and next generation sequencing (NGS) of complex DNA mixtures^4^) has been used in the field of wildlife ecology to assess a species diet and to infer prey-predator relationships or ecological networks based on faecal samples (e.g.^5–10^). Short DNA metabarcodes, usually less than 150 base pairs (bp)^11^, ideally combine high taxonomic coverage and resolution, and have the great advantage to be applicable to degraded DNA, which is the very characteristic of digested food samples. So far, the only study available on humans tested in a clinical context the methodological feasibility of using DNA metabarcoding of faecal samples to compare the inferred plant components with self-reported lists of eaten items, highlighting the potential of this approach^12^. Alternatively, the analysis of stomach content samples, although invasive, provides the advantage that aliments (and consequently DNA) are less digested and degraded than after their passage through the intestinal tract^13^. DNA metabarcoding studies of stomach content samples have already been done to assess the diet of wildlife taxa such diverse as e.g. krill^14^, Norwegian lemmings^15^, Antarctic toothfish^16^, spiders^17^, Pygmy devil rays^18^ and bugs^19^.

Despite their clear advantages, molecular approaches have surprisingly not yet been established in routine human forensics to comprehensively investigate the last meal components of deceased persons (but see^20^ in a very different context). The few studies published to date focused on the identification of a single taxon or food items (i.e. tomato and pepper seeds in faeces, mushrooms in clinical forensic samples, dandelion juice in the stomach of a presumed murder victim) using amplified fragment length polymorphism (AFLP) analysis^21,22^ or PCR followed by Sanger sequencing^23,24^. While these studies demonstrate the interest to genetically identify digested food for forensic purposes, the scope of species-specific assays remains limited and their respective development time-consuming. In addition, the use of solid or structural intact particles as a source of DNA is not always possible when dealing with (partially) digested stomach contents.

To overcome these limitations, in this study we applied for the first time a DNA metabarcoding approach to test its potential to assess both plant and vertebrate components of human stomach content samples, taken during medicolegal autopsies. We identified several plant and animal taxa, consistent with previous food consumption descriptions in the studied region. Our results suggest that this method could reveal crucial information in providing corroborative evidence about the last hours preceding death. Besides being useful for purely forensic objectives, our study opens promising perspectives in the wider context of human dietary surveys based on digested food items found in the gastrointestinal tract or in faecal samples.

## Results

**Figure 1:**
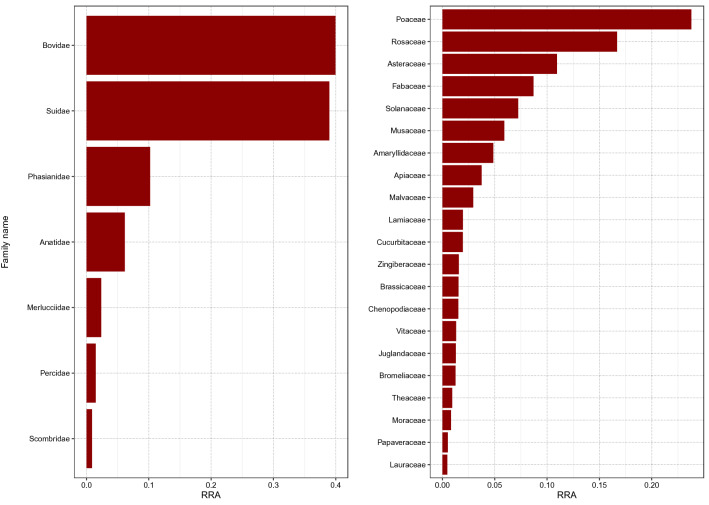
Barplot representing the sum of relative read abundances (RRA) for vertebrate (left panel) and plant (right panel) items across all samples at family level. Human reads were removed before calculating RRA scores.

**Figure 2:**
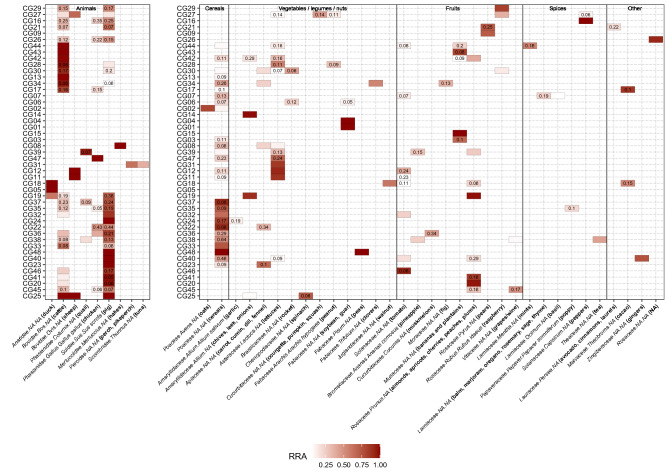
Heatmap representing relative read abundances (RRA) of detected items per sample. Values inside each box show the standard deviation of the mean between replicates. RRA scores have been calculated separately for vertebrates and plants. Within each sample, Sper01 OTUs not constituting at least 10% of RRA and Vert01 OTUs below 5% are not shown in the heatmap. We indicate Family.Genus.species assignments according to the *ecotag* command, along with the common name of all edible species or group of species (written in bold in brackets) which resulted in a 100% match with the NCBI database after manually blasting every sequence. NAs are shown in order to better visualise taxonomic resolution of the ecotag assignments.

After all quality filtering steps and merging of the data of all 48 samples, the final dataset for the Vert01 assay contained 34 different vertebrate sequences, clustered into 9 operational taxonomic units (OTUs), excluding human DNA. The Sper01 assay contained 124 different plant sequences, clustered into 33 OTUs. The relative read abundance (RRA) of animal and plant items is summarised per assay for all samples combined (Fig. 1). The heatmap shows RRA of all OTUs found per individual sample (Fig. 2). Total RRA of all OTUs per sample and replicate can be found in Supplementary Table S2.

We obtained plant sequences for all samples but one (CG16) that did not retain any OTUs after all filtering steps. We successfully amplified non-human vertebrate DNA in 34 samples, the remaining 14 resulted only in human DNA sequences.

We statistically tested for an effect of the digestion degree on the amount of different sequences retained per sample (both plants and animals) using a Pearson correlation test, but found no significant correlation (*r*(42) = 0.20, *p* = 0.18). We also tested for the effect of days since death (time between death and autopsy), but found no significant correlation (*r*(42) = 0.10, *p* = 0.49) neither for plants nor for animals.

## Discussion

In this study we successfully applied a DNA metabarcoding approach to identify consumed food items of plant and animal origin in human stomach content samples, even when digestion was advanced and macroscopic inspection no longer possible. A wide panel of common and less common edible food items were found, including meat, fish, legumes, cereals, nuts, fruits and spices. So far, gastric content analyses in a forensic context are typically based on microscopic and macroscopic identification of food items (reviewed e.g. in^1^). However, this approach is characterised by low taxonomic resolution, low sensitivity, and proves ineffective when meal leftovers are rendered unidentifiable due to chewing and digestive processes. In the field of molecular ecology, studies on animals have shown that morphological identification of prey items in the stomach underestimates prey diversity, which is particularly true when digestion is advanced (e.g.^25^). The only study to date applying DNA metabarcoding to infer human diet was based on faecal samples and did not assess any animal components of diet, although including a controlled feeding trial of an animal-based diet^12^. The comparison of the obtained plant DNA sequences to self-reporting indicated that, while some items were not reported but detected by DNA metabarcoding, all but one self-reported items were detected (the only exception being coffee), thus highlighting the sensitivity of the method. The present study, based on a random sampling of 48 human stomach contents collected during routine autopsies, includes a higher number of vegetal items and shows for the first time the successful detection of dietary items of animal origin. We found no correlation between the diversity of species detected and the time since death or digestion degree, which advocates for the utility of this methodology. The Vert01 primer set, highly specific to vertebrates, enables to distinguish between commonly eaten animal taxa and is clearly advantageous over morphological identification. In line with regional eating habits and previously published diet surveys^26^, we found within the 48 samples mainly pig, cattle/dairy and OTUs assigned to the plant families Poaceae, Rosaceae and Asteraceae (likely cereals, fruits, lettuces; Fig. 1). We did not detect coffee (*Coffea spp.*) in any of the stomach content samples, in line with^12^, which might be due to a degrading effect of roasting procedures on DNA, the absence of this popular beverage in all of the stomach samples being unlikely. Similarly, although common in Swiss eating habits, we also did not detect potato, which is usually eaten boiled or baked. Note that additional edible plant species, not listed in Fig. 2 since not constituting at least 10% of RRA but with 100% match with the database, were also detected (e.g. buckwheat, citrus fruits, flax, mangoes, sesame; Supplementary Table S2). Because we could obviously not compare our results to self-reported diets, we applied very stringent filtering parameters to avoid the occurrence of false positives (see Bioinformatic data treatment). It is beyond the approach of this study to distinguish between the animal source and a final processed food item (e.g. dairy or egg products) based on the obtained DNA sequences. However, this could be achieved by complementing the primer set with a bacterial marker (to e.g. identify the presence of a particular cheese^27^) or using proteomics (see below).

Overall, the Vert01 metabarcode is able to discriminate well among commonly eaten genera. However, owing to its limited taxonomic resolution (72.4% at the species level, based on in silico testing^11^), species-level distinction is not always possible (e.g. between perch and pikeperch) or between potentially-eaten wild species and their conspecific domestic counterparts (e.g. wild boar and pig). In Fig. 2, we present the taxonomical assignation done using *ObiTools* together with a common name, selected after manually inspecting each sequence using BLAST and only considering 100% matches with edible species. In some cases, the common name refers to a group of species because the barcode was not specific enough to distinguish between genera or species. This is more relevant concerning plants, as the Sper01 metabarcode length ranges from 10 to 220 bp, implying that some items with shorter metabarcode and/or closely related phylogenetically could not be distinguished to genus or species level due to limited resolutive power. This is related to the nature of this universal plant marker, which has been designed to target a region of the *trn*L intron of chloroplast DNA which lacks taxonomic resolution within several plant families (only 21.5% resolution at the species level^9,11^) but has wide taxonomic coverage. This trade-off meant for our study that we could genetically not distinguish between some close species which are clearly different morphologically (e.g. stone fruits, cucurbits). To overcome this issue and increase the taxonomic resolution of the results, it is possible to envisage multiplexing within the same PCR of additional primers specifically targeting groups of species that cannot be identified at the species level by the P6 loop of the trn*L* intron. Such a strategy has already been implemented to distinguish between *Carpinus betulus* and *Corylus avellana* in bison diet^28^. Furthermore, it must be outlined that by using these primer sets only, diet assessment is not comprehensive as it does not target all possibly present food products. Even so-called universal primers may result in preferential amplification of some taxa over others and non-amplification of target taxa^29,30^. For this pilot study, we chose to use two universal PCR primer pairs with wide taxonomic coverage but limited specific resolution, in order to detect a broad range of items. To gain resolution for specific vertebrate or plant taxonomic groups (e.g. fish, birds, cereals) or target taxa not covered by these primers and which could be of forensic interest (e.g. marine crustaceans and molluscs, algae, fungi), it is possible to complement Vert01 and Sper01 with additional, taxonomically-restricted PCR metabarcoding primers described in the literature (e.g.^31^; examples reviewed in^11^). Taxonomic assignation of an unknown DNA sequence strongly depends on the exhaustiveness and quality of a reference database, either public as e.g. GenBank or custom-made/local (reviewed in^32^). In case of a priori knowledge of the overall consumed diet in samples, local databases may be restrained to the expected DNA sequences, which subsequently improves taxonomic assignment. For this study we in silico compiled databases containing all possible sequences amplified by our markers, but restricted these to vertebrates and spermatophytes (i.e. seed plants), respectively.

The duration of stomach emptying has been estimated by the percentage of a meal present in a stomach^3^, but this process is influenced by several variables including the type and volume of consumed food, lifestyle and health, and can therefore last from few hours to days^2^. While one could argue that plant items usually remain longer in the stomach, our findings do not allow to draw robust conclusions about correlations of certain food items and digestion times. In order to establish hypotheses useful for time-frame estimations, additional experiments are necessary. In a controversial case of death, MS-based proteomics provided additional information through the analysis of food-derived proteins and peptides in the gastric content sampled at autopsy, indicating a last breakfast of milk and bread. While this method is certainly promising, it might reveal difficult if digestion is in an advanced stage, and has a less comprehensive scope than a DNA metabarcoding assay^33^. Furthermore, the effect of food processing techniques on DNA quality must be taken into account since cooking denatures e.g. proteins which in turn renders DNA amplification preferential to immunological approaches^1^. Different cooking treatments (variable duration of boiling, frying, baking) of tomato seeds showed that DNA extraction yielded in good quality DNA only for fresh seeds^34^, while digestion did not destroy DNA^21^. Hence, there might be an implicit bias of DNA metabarcoding to preferentially detect non-processed food (i.e. raw versus cooked). Another issue of environmental DNA-based methods is that it is not possible to distinguish between different states of food products based on DNA sequences. As mentioned before, we could not discriminate between e.g. grapes/wine, fruits/juices, beef meat/dairy products or chicken meat/eggs, since the DNA sequence of a derived product is identical to the DNA sequence of its source. While it is less common to encounter such biases for plants, mainly in cereal-derived products, it has to be taken into account when extrapolating diet patterns from DNA metabarcoding results.

Stomach content sampling is invasive, but advantageous or even required with certain animal species and in particular circumstances, including definitely the human forensic context. An advantage of stomach content over faecal samples is that food is in an early stage of digestion before passing through the pyloric sphincter into the intestines, thus the effects of inhibition by bacteria or enzymes and degradation of DNA are less significant^11,18^. While some food particles such as seeds sometimes remain identifiable, even morphologically, after passing through the digestive system^21^, others do not and the same applies to DNA which is degraded by the digestive processes taking place in the intestinal tract. In a controlled feeding experiment on insects, the detectability of food DNA in different types of dietary samples showed that regurgitates and entire animals (including stomach content) outperformed faeces regarding detectability of prey DNA^13^. While food journals in dietary surveys may contain errors or deliberate omissions^12^, they are a comprehensive and easily accessible method of human diet assessment. However, in case of deceased persons that option is no longer available.

Stomach content analyses provided crucial information for criminal investigations about cases of sudden and unexplained death on numerous occasions in recent years, enabling investigators to interpret perimortem events in detail (case examples reviewed in^2^). The results of this pilot study show that human stomach content analyses by DNA metabarcoding can be used as a complementary tool to traditional forensic macro- and microscopic approaches, with clear advantages such as an almost unlimited flexibility in terms of nature and range of taxa targeted, as well as high sensitivity and taxonomic resolution. Consequently, information that might otherwise remain undetected can be revealed, highlighting timings and circumstances surrounding the last hours of a person and his/her food intake. In a broader perspective, taking into account the potential improvements and refinements described above, and the growing amount of research literature available for wildlife species (i.e. environmental DNA-based studies), our results open up promising and novel prospects in the broader framework of human biomedical investigations of dietary patterns, based on partially or fully digested food found in the gastrointestinal tract or in faecal samples.

## Methods

### Sample collection

In this proof of concept study, we selected 48 anonymised frozen stomach content samples collected during medicolegal autopsies performed at the Lausanne University Center of Legal Medicine (Switzerland) in 2015. The inferred time span between death and autopsy was noted. For each sample, a degree of digestion as defined in^35^, ranging from 1 (no signs of digestion, mainly solid components) to 4 (complete digestion, only liquid content), was given after macroscopic inspection (Supplementary Table S1). Following gentle mixing to ensure representativity, subsamples of 45–50 mL were transferred to BMT-50-S tubes for grinding with stainless steel beads (IKA, Staufen, Germany) and stored at -20 °C until DNA extraction.

### DNA extraction

Two independent extractions per sample were performed using the DNeasy *mericon* Food Kit (Qiagen, Hilden, Germany) recommended for this sample type^35,36^. A subset of the extractions was tested for inhibitors with quantitative real-time PCR (qPCR) applying different dilutions in triplicates. qPCR reagents and conditions were the same as in DNA metabarcoding PCR reactions (see below), with the addition of 10,000 fold diluted SybrGreen (Thermo Fisher Scientific, USA). Following these analyses, all samples were diluted fivefold before PCR amplification. All extractions were performed in a laboratory restricted to forensic or low DNA-content analyses.

### DNA metabarcoding assay

In order to assess a broad range of potential food components in human diet, samples were amplified using two different primer pairs, targeting taxa of both animal and vegetal origin. The first primer pair targets a 56–132 bp gene fragment of the 12S mitochondrial DNA gene in vertebrates (Vert01^11^; corresponding to 12SV5F/R^37^), allowing the amplification of animal-derived components of human diet. A human-blocking primer^9^ that binds human DNA sequences to limit their amplification was added. As shown in a previous study^8^, the chosen concentration of the human-blocking primer (see below) corresponds to the best compromise between the efficiency of the amplification of the vertebrate species and the blocking effect over the unwanted target. The second primer pair amplifies the P6 loop of the *trn*L intron (UAA) of chloroplast DNA (10–220 bp) and targets plant components of the diet (Sper01^11^; corresponding to g/h^38^). To allow attribution of DNA sequences to samples, primers were tagged with eight variable nucleotides added to their 5′-end with at least five differences between tags. The PCR reactions were performed in a final volume of 20 µL, using 96-well plates. The mixture contained 1 U AmpliTaq Gold 360 mix (Thermo Fisher Scientific, USA), 0.04 µg of bovine serum albumin (Roche Diagnostics, Basel, Switzerland), 2 µM of human-blocking primer (coupled with Vert01 primers only), 0.2 µM of tagged forward and reverse primers and 2 µL of fivefold diluted template DNA. PCR cycling conditions were denaturation for 10 min at 95 °C, followed by 40 cycles of 30 s at 95 °C, 30 s at 49 °C (Vert01) or 52 °C (Sper01), and 1 min at 72 °C, with a final elongation step of 7 min at 72 °C. For each assay, we included: (i) extraction negative controls; (ii) PCR negative and positive controls; (iii) blanks. Blanks correspond to empty wells on the PCR plate (i.e. no primer, no template) enabling to estimate the percentage of tag switches^39^. Both DNA extraction duplicates were amplified in triplicate (i.e. six PCR amplifications per sample were performed in total). Amplification success and fragment sizes were confirmed on a 2% agarose gel. Amplicons were pooled per plate, purified using a MinElute PCR Purification Kit (Qiagen, Hilden, Germany) and quantified using a Qubit 2.0 Fluorometer (Life Technologies Corporation, USA).

Two sequencing runs were performed, the first one to test the method on 12 samples (CG01-CG04, CG06-CG12, CG14), the second run including the remaining 36 samples. For the first run, library preparation and sequencing were performed at Fasteris facilities (Geneva, Switzerland). Libraries were prepared using the Metafast protocol (https://www.fasteris.com). A paired-end sequencing was carried out in an Illumina HiSeq 2500 (2 × 125 bp; Illumina, San Diego, CA, USA) using the HiSeq SBS Kit v4 (Illumina, San Diego, CA, USA) following the manufacturer’s instructions. For the second run, library preparation was performed using the TruSeq DNA PCR-Free Library Prep Kit (Illumina, San Diego, CA, USA) with an adjusted beads ratio of 1.8 to remove small fragments. After adapter ligation, libraries were validated on a fragment analyser (Advanced Analytical Technologies, USA). Final libraries were quantified, normalised and pooled before 150 paired-end sequencing on an Illumina MiniSeq sequencing system with a Mid Output Kit (Illumina, San Diego, CA, USA).

### Bioinformatic data treatment

The bioinformatic processing of the raw sequences output was performed using the *ObiTools* package^40^. The following steps were done separately for each library (i.e. per PCR plate containing each 12 samples and controls). Initially, forward and reverse reads were assembled with a minimum quality score of 40. The joined sequences were assigned to samples based on unique tag and primer combinations allowing two mismatches on primers and no mismatches on tags. Assigned sequences were then dereplicated, retaining only unique sequences. All sequences with less than 100 reads per library were discarded as well as those not fitting the above stated metabarcode lengths. This was followed by two different clustering methods. First, pairwise dissimilarities between reads were computed with the *obiclean* command and lesser abundant sequences with single nucleotide dissimilarity were clustered into the most abundant ones^40^. Second, we used the *sumaclust* algorithm^41^ to further refine the resulting clusters based on a sequence similarity of 97%. Using the program ecoPCR^42^ on the EMBL 2019 release, we built two databases by running in silico PCRs based on primer sequences and expected metabarcode lengths for Vert01 (16,292 sequences, Supplementary Data S1) and Sper01 (18,636 sequences, Supplementary Data S2). These databases were restricted to vertebrate and spermatophyte taxa, respectively. Sequences were assigned to taxa present in the database using the *ecotag* command, with a similarity threshold of 97%. Operational taxonomic units (OTUs) with similarity lower than 97% were eliminated from the dataset.

Further data cleaning and filtering was done in R (version 3.6.2). Sequences that were more abundant in extraction and PCR controls than in samples were considered as contamination and removed. To account for tag switching, we considered the leaking of a sequence to be directly linked to its abundance. To test this, we performed Wilcoxon signed-rank tests between samples and blanks and consequently removed from all samples a given ratio of presumed tag-leaked sequences. Dysfunctional PCR replicates were also discarded, i.e. with too small overall reads count based on library-dependant thresholds^11^. Final count of reads was transformed to RRA in order to have a normalised and comparable dataset between samples and sequencing runs. In the next step, PCR replicates were merged by sequence and extraction. Sequences that were present in only one out of three PCR replicates were removed, in line with^42^. This approach allowed us to discard single OTUs instead of whole PCR replicates. Finally, we combined the extraction duplicates of a sample, and calculated the mean count per OTU for each sample as well as the standard deviation.

For this resulting dataset (124 plant and 34 animal sequences), we re-assessed the taxonomic assignment done by *ObiTools (ecotag)* in order to assign a common name to each OTU, acknowledging in particular the limitations of the Sper01 metabarcode for domesticated varieties that share identical sequences for the *trn*L-P6 locus. We blasted^43^ each sequence (Sper01 and Vert01) on the NCBI database and compared the results with the *ecotag* assignments.

### Ethical statement

Study protocol was approved by the *Cantonal Commission on Ethics in Human Research* (Lausanne, Switzerland). Since the analyses do not concern the human genome but only aim at amplifying and analysing animal and plant DNA for research purposes, and since samples were completely anonymised, the study protocol does not require informed consent.

## Supplementary Information

Supplementary Information

## Data Availability

The datasets analysed during the current study are available from the corresponding author on reasonable request.
